# Enzymatic Treatment of Specimens before DNA Extraction Directly Influences Molecular Detection of Infectious Agents

**DOI:** 10.1371/journal.pone.0094886

**Published:** 2014-06-17

**Authors:** Pablo Goldschmidt, Sandrine Degorge, Lilia Merabet, Christine Chaumeil

**Affiliations:** Laboratoire du Centre Hospitalier National d'Ophtalmologie des Quinze-Vingts, Paris, France; University of California, San Francisco, University of California, Berkeley, and the Children's Hospital Oakland Research Institute, United States of America

## Abstract

**Introduction:**

Biological samples, pharmaceuticals or food contain proteins, lipids, polymers, ammoniums and macromolecules that alter the detection of infectious agents by DNA amplification techniques (PCR). Moreover the targeted DNA has to be released from the complex cell walls and the compact nucleoprotein matrixes and cleared from potential inhibitors. The goal of the present work was to assess the efficiency of enzymatic pretreatments on infectious agents to make DNA available for further extraction and amplification.

**Methods:**

*Staphylococcus epidermidis*, *Streptococcus mitis*, *Propionibacterium acnes*, *Escherichia coli*, *Pseudomonas aeruginosa*, *Candida albicans*, *Aspergillus niger and Fusarium solani* were mixed with an internal control virus and treated with: 1) proteinase K; 2) lyticase and 3) lyticase followed by proteinase K. DNAs was manually extracted using the QIAmp DNA Mini kit or the MagNA Pure Compact automate. DNA extraction yields and the inhibitors were assessed with a phocid *Herpesvirus*. Bacterial detection was performed using TaqMan real-time PCR and yeasts and filamentous *Fungi* with HRM (real-time PCR followed by high-resolution melting analysis).

**Results:**

Viral DNA was released, extracted and detected using manual and automatic methods without pre enzymatic treatments. Either the manual or the automatic DNA extraction systems did not meet the sensitivity expectations if enzymatic treatments were not performed before: lyticase for *Fungi* and Proteinase K for *Bacteria*. The addition of lyticase and proteinase K did not improve results. For *Fungi* the detection after lyticase was higher than for Proteinase K, for which melting analysis did not allow fungal specification.

**Discussion:**

Columns and magnetic beads allowed collecting DNA and separate PCR inhibitors. Detection rates cannot be related to DNA-avidity of beads or to elution but to the lack of proteolysis.

## Introduction

In situations where rapid diagnostic decisions are required, the culture methods (gold standard) take several hours to days to yield results and may produce erroneous results for fastidious species [1]. The culture performances depend among other factors on the type of agent, the microbial load, the mass of material that can be processed, the residual level of antibiotics, antiseptics or antifungals and the microbiology laboratory capacities [2], [3]. Timely diagnosis allows rapid onset of targeted treatments [4]–[8] but at least 12-h to 48-h of incubation followed by several assays to identify the agent are necessary to obtain a positive conclusion. Time for diagnosis is even longer if antibiotherapy was started before sampling or for slowly growing organisms [1], [2], [9], [10]. For fungal and bacterial infections, approximately one half of the samples remain culture negative and/or negative by fungal antigen detection using immunoenzymatic assays (ELISA) [11]–[13].

The nucleic-acid amplification based tests (NAATs) show higher sensitivity than cultures and are less affected by the prior use of antibiotics or antifungal agents [14], [15]. However, the tests based on the classic polymerase chain reaction technology (PCR) are unable to differentiate among species and require post amplification procedures (restriction enzyme digestion and analysis; single-base extension; hybridization probes or molecular sequencing) [7], [8], [16], [17]. Species characterization may require DNA sequencing (laborious, expensive and difficult to be performed on primary samples for daily diagnosis in clinical sets) [15]–[18]. To improve microbiology diagnosis and sterility testing directly from samples we developed rapid molecular approaches producing in only one run a series of quantifiable signals, in which relevance is automatically interpreted. These tests are carried out in an environment kept closed after the DNA extraction [19]–[21].

Biological, environmental, pharmaceuticals or food samples generally contain proteins, hyaluronic acid, lipids, polysaccharides, heparin derivatives, quaternary ammoniums, inorganic compounds or several macromolecules that may alter NAAT performances (inhibiting the Taq polymerase they induce false negative results). Therefore, the DNA has to be released from the primary samples and from the complex cell walls and the compact nucleoprotein matrixes to be cleared from potential PCR inhibitors and extracted [20]–[22].

The manual methods for the extraction (purification) of nucleic acids from urine, serum, blood, skin, bones, corneal scrapings, aqueous and humor and vitreous and from cultures are laborious and time consuming: many steps involve detergent mediated lysis, enzymatic and/or chaoptropic treatment, organic solvent extraction and further precipitation and sediment washing and concentration [22]–[24].

A major contribution for the extraction of DNA was reported by associating lytic enzymes with the physicochemical properties of the extraction solutions and of chaoptropic agents to release nucleic acids, allowing them to bind to the surface of ferrous active beads [25].

In previous studies we showed that rigid *Acanthamoeba* cysts are resistant to reagents releasing DNA from mammalian cells and viruses (heat, NaOH, proteinase K). However, a significant increase in positive results (without affecting the DNA detection rates) was observed by a proteinase K pretreatment (10 min at 56 degrees C) before the automatic commercial extractive procedures (MagNA Pure or QIAmp) [28].

Considering that the impact on the overall diagnosis sensitivity is associated with the DNA extraction technology, diagnosis approaches should recover very small amounts of DNA from clinical specimens [26]–[28]. In situations in which viral, bacterial, parasitic and fungal DNA has to be extracted from the same specimen, isolated enzymatic treatments seem not efficient enough to warrant the highest performances [27]–[31]. The goal of the present work was to assess the efficiency of enzymatic treatments according to their ability to make DNA available for automatic magnetic bead extraction. The efficacy of three pre-treatments was studied for quantified preparations of *Streptococcus mitis*, *Staphylococcus epidermidis*, *Escherichia coli*, *Pseudomonas aeruginosa*, *Propionibacterium acnes*, *Aspergillus niger*, *Candida albicans*, *Fusarium solani*, and a phocid *Herpesvirus*.

## Methods

### Ethic Statements

All research involving human samples has been approved by the Institutional Review Board (IRB) at the Centre Hospitalier National d'Ophtalmologie de Paris (CHNO). All the investigations have been conducted according to the principles expressed in the Declaration of Helsinki (http://www.wma.net/e/policy/). The patient written consent forms were obtained before sampling for diagnosis purposes and for use of residues or of isolated strains for improving the knowledge. The written consent forms drafted according to the requirements of the IRB and the National Health Authorities were sent to the laboratory after being double checked, validated, and signed by the physician in charge of the sampling. Consent forms are stocked at the archives of the CHNO and according to the CNIL (*Commission Nationale Informatique et Libertés*) these are not public documents. Research was performed with characterized bacterial and fungal strains isolated from patients presenting corneal ulcers and data were analyzed anonymously.

### Preparation of quantified bacterial and fungal suspensions

To reduce the over representation of DNA from non-viable microorganisms, one colony of *Staphylococcus epidermidis*, *Streptococcus mitis*, *Propionibacterium acnes*, *Escherichia coli*, *Pseudomonas aeruginosa*, *Candida albicans*, *Aspergillus niger and Fusarium solani* was scraped from the agar plates, suspended and re plated on a solid rich-agar (*Fungi* were plated on Sabouraud's solid agar). After 24 hours, one colony was scraped from the second plate, suspended in Phosphate Buffer Solution (PBS) and tenfold diluted. Each dilution was divided in several aliquots; three were plated on agar to assess the number of colonies (equivalent CFU/ml) and the others kept as calibrators. Randomized tubes containing saline were spiked with titrated bacterial or fungal suspensions (10^2^ to 10^4^ CFU/ml).

### DNA extraction procedures

The DNA extraction procedures were carried out in a vertical safety laminar flow cabinet in a dedicated room. To monitor the extraction yields and the absence of PCR inhibitors the internal control (IC, high molecular-mass tracer) consisting of 5 µl of a whole virus (gift from G. J. van Doornum, Dept. of Virology Erasmus MC, Rotterdam, The Netherlands) was added to each suspension before extraction (final concentration of 1000 to 2000 viral particles/ml) [19]–[21], [30], [31]. Each reaction was validated if the differences in the Ct values for the IC for each sample were <2.0 respect to the value for the IC suspended in saline. For the 3 pre-treatments the final volume (specimen + enzymes + buffer) was homogenized (300 µl) and 100 µl were extracted.

### Proteinase K treatment

Proteinase K (QIAgen, France) is a serine protease that cleaves the peptide bond adjacent to the carboxyl group of aliphatic and aromatic amino acids with blocked alpha amino groups [32]. Each suspension (sample + IC) was mixed with tris-EDTA buffer (inhibition of calcium-dependent nucleases) and 100 µl of proteinase K and incubated at 37°C for 60 min and heated at 94°C for 10 min to inactivate the enzyme. One hundred µl were used for DNA extraction [20].

### Lyticase treatment

Lyticase (Sigma-Aldrich, France) is a synergistic enzyme complex of endoglucanase and protease that catalyzes yeast cell lysis by *β*-1,3-glucanase (linear glucose polymers at beta.-1,3-linkages) and a highly specific alkaline protease activity, producing protoplasts or spheroplasts [33].

Bacterial or fungal suspensions (samples + IC) were mixed with tris-EDTA buffer and 10 U recombinant lyticase/100 µl of suspension and incubated at 37°C for 60 min. The suspensions were vortexed thoroughly, heated for 10 min at 94°C, cooled and 100 µl were used for DNA extraction [30], [34].

### Proteinase K followed by Lyticase treatment

Each specimen (sample + IC) was mixed with tris-EDTA buffer and 10 U recombinant lyticase (Sigma-Aldrich, France)/100 µl of suspension and incubated at 37°C for 60 min. After incubation, the suspensions were vortexed thoroughly, heated for 10 min at 94°C and after cooling at room temperature 100 µl were mixed with tris-EDTA buffer and 100 µl of proteinase K and incubated at 37°C for 60 min and heated at 94°C for 10 min to inactivate the enzyme and extracted.

### DNA extraction procedures

#### QIAmp DNA Mini kit and MagNA Pure Nucleic Acid isolation kit

The DNA extraction was performed manually using the solid column-based extraction kit QIAmp DNA Mini kit tissue protocol (Qiagen, France) or by magnetic beads using the MagNA Pure Nucleic Acid isolation kit with the MagNA Pure Compact (Roche) automate. Nucleic acids were eluted in 100 µl of DNA free distilled-water. The time needed for the manual QIAmp procedure was 120 min with no additional equipment required and for MagNA Pure less than 28 min with a specific dedicated robot.

#### Detection and characterization of *Bacteria*

Bacterial detection was performed using real-time PCR with primers chosen from regions of identity within the 16S rDNA following the alignment of sequences outlined in Bergey's Manual of Determinative Bacteriology [35], [36]. Testing was carried out by adding 10 µl of the extracted DNA to each of the 4 tubes containing the primers (0.5 uM) and fluorophore-labelled TaqMan probes (0.5 uM) in 10 µl of TaqMan FAST Universal PCR Mastermix (Applied Biosystems-France Ref. 4352042). The cycling program consisted in 1 cycle at 95°C for 10 min and 45 cycles of amplification (15 s at 95°C, 8 s at 52°C, and 10 s at 72°C). Each run contained negative controls with no template and DNA extracts from the reactants [20], [21].

#### Rates of extraction of DNA and detection of Taq polymerase inhibitors

The extraction yields of DNA and the polymerase inhibitors were assessed using a phocid *Herpesvirus* as internal control (IC) (for both the TaqMan real-time PCR for *Bacteria* and the HRM for *Fungi*). If more than one probe had to be introduced into one reaction tube (for real-time PCR), the differences in the emission wavelength spectrums of the fluorophores were separated in the spectra at least for 15 nanometers. The IC primer sequences were: 5′GGGCGAATCACAGATTGAATC and 5′GCGGTTCCAAACGTACCAA and VIC-TTTTTATGTGTCCGCCACCATCTGGATC-TAMRA for the probe [20], [30], [31].

#### Detection of yeasts and filamentous *Fungi* by HRM (real-time PCR with high resolution melting analysis)

The selected primers bracket significant polymorphisms of multicopy ribosomal genes of the 18S ribosomal RNA gene [30], [36]–[38]. The Primer 1: HRM CandUn1: 5′CATGCCTGTTTGAGCGTC (conserved sequences of yeasts,) and the Primer 2: HRM FungUn: 5′TCCTCCGCTTATTGATA TGCT (conserved regions of all *Fungi*) allow obtaining profiles for the different yeasts according to the sizes of amplicons (alignment of sequences according to EMBL data library). The amplicon size for *Candida albicans* (nucleotides bracketed by the primers CandUn + FungUn) is 189. The primer sequences for filamentous *Fungi* produce amplicons of 180 to 210 nucleotides. The selected sequences are: HRM FilamUn: 5′TGCCTGTTCCGAGCGTCAT (forward primer) and HRM FungUn: 5′TCCTCCGCTTATTGATATGCT [30], [38], [39].

For HRM 10 µl of DNA extract were introduced in 2 tubes, the first containing CandUn + FungUn in the Master Mix for HRM (MMHRM, Applied Biosystems, France) and the second FilamUn + FungUn. The neo formed amplicons were measured in a closed tube format using integrated cycler/fluorometer ABI 7500 upgraded equipment and monitored using the fluorescent SYTO9 DNA intercalating dye present in the MMHRM. The PCR program started with a denaturation of 10 min at 95°C, followed by 55 cycles of amplification (15 s at 95°C, 30 s at 60°C and 30 s at 72°C). The PCR-HRM curve was drafted after denaturation at 95°C for 15 sec, cooling to 50°C for 1 min and a temperature increase until 60°C for 15 sec with a 2.2°C/s ramp rate. Samples with fluorescence of less than the 100% of the maximum were excluded from the analysis. The melting temperature (Tm) at which 50% of the DNA is in the double stranded state was assessed by taking the derivative of the melting curve. The DNA patterns of the derivative plot (difference plot) were used for amplicon analysis [38].

For HRM the additional IC detection was carried out in a dedicated tube containing 18.5 µl of the TaqMan FAST Universal PCR Mastermix (2× no Amperase UNG) (Applied Biosystems-France ABI Ref. 4352042), the forward and the reverse primers (0.5 uM each) with or without the fluorophore-labelled TaqMan probe (0.5 uM). This solution was mixed with 5 µl of the DNA eluted in DNA and RNA-free solution. The PCR cycling program consisted of one cycle at 95°C for 20 sec and 45 cycles at 95°C for 3 sec and 30 sec at 60°C [39].

## Results

The Cts for the ICs indicate viral DNA was released and extracted using the manual solid column-based extraction kit QIAmp and the MagNA Pure Nucleic Acid isolation kit (Roche) with the MagNA Pure Compact (Roche) automate. For the 2 methods pre enzymatic treatments did not improve the rate of detection of the phocid *Herpesvirus* used as IC. These results confirm that both extraction systems eliminate the potential PCR inhibitors for agents suspended in saline [20], [30]. Surprisingly, the different enzymatic pretreatments show that the IC Cts were almost identical for all (virus) but not for *Bacteria* or *Fungi*, for which the Cts were significantly different depending from the pretreatment before magnetic bead automatic DNA extraction (differences >3 Cts).

In Table S1 and Table S2 are presented the results after 3 different enzymatic pre-treatments on spiked suspensions. For the lowest bacterial and fungal loads (equivalent to 10^2^ CFU/ml) neither the manual nor the automatic DNA extraction systems meet the highest sensitivity expectations if lyticase (to transform rigid yeast and spores in fragilized spheroplasts [33], [43]) or proteinase K pretreatments were not carried out respectively for *Fungi* or for *Bacteria.*

The pretreatment of specimens with lyticase produced low detection real-time PCR rates for several *Bacteria* at concentrations of 10^2^ CFU/ml using the universal BactUn probe [17], [20]. The performances were repeatedly reduced for 5 *Bacteria* pre-treated with lyticase, for which Cts were delayed for more than 3 cycles (>1 log PFU/ml) for *Streptococci*, *Staphylococ*ci, *Propionibacteria*, *E. coli* and *P. aeruginosa*. The lack of efficiency of lyticase treatment was confirmed with the specific probes for *Enterobacteria*, for Gram positive cocci and for 4 different *Genera* [20]. The addition of a lyticase to the proteinase K pre-treatment K did not improve *Bacteria* detection using Taqman real-time PCR.

On the other hand, fungal detection was conducted by simultaneous amplification of DNA extracts in 2 tubes, the first with the set of primers CandUn + FungUn and the second with FilamUn + FungUn. The patterns of the first derivative (difference plot) permitted differentiation of yeasts from filamentous *Fungi*. For *Candida albicans*, *Aspergillus niger* and *Fusarium solani* the detection limits were significantly reduced after proteinase K pre-treatment of compared to those achieved after lyticase (Table S2). Moreover, during the amplification process the specimens spiked with *Fungi* and treated with proteinase K produced signals that could suggest that *Fungi* were present (at concentrations of 10^3^ CFU/ml or higher) but the melting curve analysis did not allow their specification (identification) [37]–[39].

## Discussion

The results of this study confirm that a) that the Taqman real-time PCR is able to detect and semi quantify *Bacteria* and differentiates among several *Genera* [20], [31], and b) the automatic melting analysis of fungal sequences amplified with 2 sets of primers (HRM) allows detection and semi quantification of *Fungi* [39]. Both approaches were performed without post amplification procedures (gel electrophoresis, hybridizations or immunoenzymatic assays, sequencing, amplicon restriction enzyme analysis, etc.).

For fungal detection the patterns of the first (difference plot) dye-melting derivative curve analysis drafted profiles validated only if the Ct values for the IC compared to the blank were not delayed for more than 2.0 cycles.

One of the major limitations firstly describe for the diagnosis techniques based on NAATs was the inhibition of the amplification process by substances in the samples [40], [41]. Therefore, all the specimens were spiked with a known amount of a phocid *Herpesvirus* (IC) [42]. The IC signals (TaqMan real-time PCR) triggered by the IC in the bacterial or fungal suspensions were identical to their respective controls (phocid *Herpesvirus* diluted in PBS and extracted). Both, the column and the Magnetic silica bead-based strategies allowed collecting DNA and separate the PCR inhibitors and both, lyticase and proteinase K treatments before extraction produced satisfactory results for the extracted IC. On the above, the differences in *Bacteria* and *Fungi* detection cannot be directly associated to the avidity of the magnetic beads or to the intrinsic capacity of the solid columns to bind DNA, nor to the inefficiency of the elution procedures. Hence, the differences in detection rates suggest that the enzymatic pretreatments with proteinase K are not efficient for detecting low amounts of *Fungi* and the pretreatments with lyticase are insufficient to release DNA from *Bacteria* (the signals not detected represent potential risks for false negative results) [28].

Several studies evaluated manual the methods for the extraction of DNA from *Bacteria* (QIAmp Blood kit, Roche high PCR template, Puregene, boiling, glass beads/sonication and wash/alkali/heat lysis) and showed that the wash/alkali/heat lysis method was sensitive enough, reproducible and cost effective, and did remove the PCR inhibitors [43]. For intracellular *Bacteria* (*Chlamydia trachomatis*) four manual DNA extraction techniques: a) 65°C phenol, b) incubation at 97°C, c) proteinase K and d) extraction and elution using the QIAmp tissue kit, showed that the digestion with proteinase K and the heat denaturation were unable to eliminate PCR inhibitors and that only a proteinase K pretreatment followed by QIAmp extraction or by MagNApure or the hot phenol extraction produced high detection rates [19], [22].

For EDTA-human whole blood spiked with *Bacteria* the DNA recovery capacities of several commercial kits (manual QIAamp DNA Mini kit and the High Pure PCR Template Preparation kit, and two automated systems with magnetic beads (MagNA Pure LC with the DNA Isolation Kit I, and the QIAcube using the QIAamp DNA Mini kit and the QIAamp DNA Blood Mini kit) were studied. Without enzymatic pretreatment the QIAamp DNA Mini, the MagNA Pure Compact, and the QIAcube running the QIAamp DNA Mini and QIAamp DNA Blood Mini produced detectable signals for blood spiked with 5.5×10^4^ CFU/ml of *Fungi* [43]–[46]. However, in the present study, the treatment with proteinase K followed by the extraction procedures allowed consistently detecting 10^2^ CFU/ml of *Bacteria* and *Fungi* suspended in saline.

The performances of seven different methods for manual DNA extraction from *Candida*, *Aspergillus* and *Cryptococcus sp*. have been studied for :1-lyticase and QIAgen column; 2- the lyticase step replaced by glass beads + QIAgen column (30 s at maximum speed in a bead beater followed by centrifugation at full speed for 10 min; 3-MasterPure yeast DNA purification kit (Epicentre, US); 4- benzyl alcohol/guanidine hydrochloride (BAGH) DNA extraction [3]; 5- gene trapping by liquid extraction (DrGenTle, Takara Bio Japan) and extra phenol clean-up steps; 6-yeast DNA extraction reagent Y-DER (Pierce Biotechnology, US) with the addition of linear acrylamide (Ambion, US) before 2-propanol precipitation, and 7- YeaStar genomic DNA zymolase kit (Zimo Research US), followed by spin-column purification. The manual DNA extraction by either the guanidium isothiocyanate-silica method [42] or by the QIAmp tissue kit yielded the best results if minibead beating treatments were carried out before extraction to mechanically disrupt the yeast cell walls [25], [26], [43], [44]. For cell suspensions, cotton, foam and polyester swabs spiked with *Fungi* the performances of the automated and manual DNA extraction methods were compared (automated MagNA Pure Compact and QIAcube, and the manual IT 1-2-3 DNA sample purification kit, the MasterPure Complete DNA and RNA purification kit, the QIAamp DNA blood mini kit, and the UltraClean Microbial DNA isolation kit). The results were similar for DNA extracted with both automated methods and with the manual MasterPure and QIAamp, indicating that automated extractions are suitable alternatives to laborious methods for the routine isolation of DNA [45].

In this study the stringent procedures for specimen processing and the technological contribution of the real-time PCR and the HRM (tubes kept closed during the whole procedure of amplification and during signal analysis) minimized the risks for cross contamination and false positive conclusions [15], [20], [28]. On the other hand, it could have been expected that Taqman real time PCR and HRM also minimized the risks for false negative results (these techniques are extremely sensitive and the yields of extraction of DNA were assessed by systematic testing the IC introduced in all the specimens before DNA extraction) [20]. However, viruses, free DNA or plasmids used for both manual and automatic DNA extraction procedures appear incapable to reveal the real performances of the extraction procedures. On the above, it appears that NAATs require comprehensive evaluations to proof for samples with low infectious loads that the targeted DNA is available for its amplification after being extracted from the compact matrixes of the different infectious agents and from the tissues [47]–[48]. The picture of patients with fungaemia, in which the fungal load in blood can be as low as 1 CFU/ml blood enforces the requirement for systematic optimization of DNA extraction [23], [49], [50]–[52].

The first-line therapy for Gram-positive and Gram-negative *Bacteria* or for yeasts and filamentous *Fungi* as well as for different yeasts, viruses and parasites is different, and in several clinical situations the highly sensitive and specific diagnosis tests that have to be performed on only one sample may orientate treatments according to the susceptibilities of the detected agents [52]–[55]. Nevertheless, in clinical environments the potential false negative results frustrate diagnosis and laboratories cannot always count with systematic double checking of PCR results by asking additional samplings (especially central spinal fluid, corneal scrapings, intraocular fluids and biopsies).

So far, for the molecular detection of infectious agents there is no universal enzymatic treatment that warrants optimal DNA release for magnetic bead or column extraction. The material to be tested requires 2 different pretreatments before DNA extraction (manual or automatic) because the highest detection rates for *Bacteria* and for *Protozoa* (*Acanthamoeba*) [28] are achieved after pretreating samples with proteinase K and with lyticase for *Fungi*. Finally, the optimization diagnosis performances implies a relevant increase in the cost of the DNA extraction procedures because 2 separate devices are necessary, one for DNA extraction after proteinase K and the other after lyticase treatment.

## Supporting Information

Table S1 Polymerase chain reaction (PCR) results of suspensions spiked with *Bacteria* after treatment with proteinase K and/or lyticase.
(DOC)

Table S2 High resolution melting real-time PCR (HRM) results of suspensions spiked with yeast or filamentous *Fungi* after treatment with proteinase K and/or lyticase.
(DOC)
