# Supplementary material for: Enzymatic Treatment of Specimens before DNA Extraction Directly Influences Molecular Detection of Infectious Agents
Source: PLoS One. 2014 Jun 17;9(6):e94886. doi: 10.1371/journal.pone.0094886 (PMC4061000; doi:10.1371/journal.pone.0094886)
Supplement: Table S1 — Polymerase chain reaction (PCR) results of suspensions spiked with Bacteria after treatment with proteinase K and/or lyticase. (DOC) [file pone.0094886.s001.doc]

**Table S1:** CFU: colony forming units assessed after 24 h of culture of fresh suspensions; STr: specimen treatment; pIC: probe for IC; PK: proteinase K at 56° for 10 min, cooling at -18C for 5 min and extraction with magnetic beads; Ly: Lyticase at 56° for 10 min, cooling at -18C for 5 min and extraction with magnetic beads; IC: internal control; pIC: probe for IC; BactUn: probe for all *Bacteria* (Universal detection); Staph: *Staphylococcus* *epidermidis*; Strep: *Streptococcus mitis*; Ente: *Enterobacteria;* Gr+: Gram positive cocci; Propi: *Propionibacterium acnes*; E.coli: *Escherichia coli*; Psar: *Pseudomonas aeruginosa*; Anig: *Aspergillus niger*; Calb: *Candida albicans*, Fus: *Fusarium solani*; NC: negative control; -:Negative, Ct > 42; Nrs: no relevant signal; MP: MagNA Pure compact (Roche) with no specimen pretreatment; QIA (QIAamp DNA Mini kit QIAgen) with no specimen pretreatment.

| Agent | [CFU/ml] | STr | *Bacteria* Taqman Real-time PCR/ Ct according to probes for | | | | | | | |
| --- | --- | --- | --- | --- | --- | --- | --- | --- | --- | --- |
|  |  |  | pIC | BactUn | Staph | Strep | Psar | Ente | G+c | Propi |
| Strep | 10^4^ | PK | 30.0 | 21.8 | - | 25.9 | - |  | 29.4 | - |
|  |  | LY | 30.3 | 33.9 | - | 33.0 | - | - | 33.9 | - |
|  |  | PK+Ly | 29.1 | 22.0 | - | 26.9 | - | - | 29.6 | - |
|  | 10^3^ | PK | 30.1 | 25.9 | - | 29.1 | - | - | 34.0 | - |
|  |  | LY | 31.1 | 35.7 | - | 34.1 | - | - | - | - |
|  |  | PK+Ly | 29.5 | 26.1 | - | 29.2 | - | - | 34.1 | - |
|  | 10^2^ | PK | 30.5 | 30.0 | - | 33.5 | - | - | 40.9- | - |
|  |  | LY | 31.0 | - | - | 37.7 | - | - | - | - |
|  |  | PK+Ly | 29.5 | 30.1 | - | 33.3 | - | - | 41.9- | - |
|  |  | MP | 29.4 | 39.0 | - | - | - | - | - | - |
|  |  | QIA | 33.1 | 42.0 | - | - | - | - | - | - |
| Propi | 10^4^ | PK | 30.3 | 21.8 | - | - | - | - |  | 28.9 |
|  |  | LY | 28.7 | 34.8 | - | - | - | - |  | 33.4 |
|  |  | PK+Ly | 29.9 | 21.0 | - | - | - | - |  | 29.1 |
|  | 10^3^ | PK | 31.0 | 25.3 | - | - | - | - |  | 32.9 |
|  |  | LY | 29.1 | 37.0 | - | - | - | - |  | 37.9 |
|  |  | PK+Ly | 28.9 | 25.0 | - | - | - | - |  | 32.0 |
|  | 10^2^ | PK | 30.5 | 30.1 | - | - | - | - |  | 36.4 |
|  |  | LY | 28.8 | - | - | - | - | - |  | 40.8 |
|  |  | PK+Ly | 29.6 | 30.0 | - | - | - | - |  | 36.7 |
|  |  | MP | 30.0 | - | - | -- | - | - | - | - |
|  |  | QIA | 29.8 | - | - | - | - | - | - | - |
| Staph | 10^4^ | PK | 31.9 | 25.7 | 30.4 | - | - | - | 25.7 | - |
|  |  | LY | 32.9 | 32.5 | 36.1 | - | - | - | 28.0 | - |
|  |  | PK+Ly | 32.2 | 25.9 | 30.9 | - | - | - | 26.1 | - |
|  | 10^3^ | PK | 29.9 | 28.8 | 33.9 | - | - | - | 29.0 | - |
|  |  | LY | 31.2 | 36.0 | 38.9 | - | - | - | 36.5 | - |
|  |  | PK+Ly | 31.7 | 29.0 | 31.7 | - | - | - | 37.2 | - |
|  | 10^2^ | PK | 29.1 | 32.9 | 39.9 | - | - | - | 33.9 | - |
|  |  | LY | 31.0 | - | - | - | - | - | 36.0 | - |
|  |  | PK+Ly | 29.5 | 32.2 | 40.0 | - | - | - | 32.1 | - |
|  |  | MP | 29.5 | 42.1 | - | - | - | - | 42.0 |  |
|  |  | QIA | 30.1 | - | - | - | - | - | - | - |
| *E.coli* | 10^4^ | PK | 30.4 | 29.7 | - | - | - | 29.5 | - | - |
|  |  | LY | 28.9 | 34.6 | - | - | - | 33.8 | - | - |
|  |  | PK+Ly | 29.6 | 29.6 | - | - | - | 30.5 | - | - |
|  | 10^3^ | PK | 30.8 | 33.0 | - | - | - | 33.7 | - | - |
|  |  | LY | 29.2 | 37.9 | - | -- | - | 35.9 | - | - |
|  |  | PK+Ly | 29.8 | 32.5 | - | - | - | 33.1 | - | - |
|  | 10^2^ | PK | 30.5 | 36.9 | - | - | - | 37.7 | - | - |
|  |  | LY | 28.7 | - | - | - | - | 41.8 | - | - |
|  |  | PK+Ly | 28.0 | 37.9 | - | - | - | 38.1 | - | - |
|  |  | MP | 28.1 | 42.2 | - | - | - | - | - | - |
|  |  | QIA | 29.1 | 42.0 | - | - | - | - | - | - |
| Psa | 10^4^ | PK | 30.8 | 28.5 | - | - | 25.6 | - | - | - |
|  |  | LY | 28.9 | 40.9 | - | - | 34.9 | - | - | - |
|  |  | PK+Ly | 28.0 | 28.1 | - | - | 25.6 | - | - | - |
|  | 10^3^ | PK | 30.1 | 32.0 | -- | - | 29.1 | - | - | - |
|  |  | LY | 29.2 | 42.0 | - | - | 38.7 | - | - | - |
|  |  | PK+Ly | 28.8 | 32.3 | - | - | 28.1 | - | - | - |
|  | 10^2^ | PK | 28.8 | 36.5 | - | - | 30.4 | - | - | - |
|  |  | LY | 28.7 | - | - | - | 40.6 | - | - | - |
|  |  | PK+Ly | 30.0 | 36.6 | - | - | 30.0 | - | - | - |
|  |  | MP | 28.9 | 42.0 | - | - | - | - | - | - |
|  |  | QIA | 30.0 | - | - | - | - | - | - | - |
| NC | | PK | 28.5 | - | - | - | - | - | - | - |
|  |  | LY | 27.9 | - | - | - | - | - | - | - |
|  |  | PK+Ly | 28.9 | - | - | - | - | - | - | - |
|  |  | MP | 29.9 | - | - | - | - | - | - | - |
|  |  | QIA | 28.9 | - | - | - | - | - | - | - |
