# Supplementary material for: Enzymatic Treatment of Specimens before DNA Extraction Directly Influences Molecular Detection of Infectious Agents
Source: PLoS One. 2014 Jun 17;9(6):e94886. doi: 10.1371/journal.pone.0094886 (PMC4061000; doi:10.1371/journal.pone.0094886)
Supplement: Table S2 — High resolution melting real-time PCR (HRM) results of suspensions spiked with yeast or filamentous Fungi after treatment with proteinase K and/or lyticase. (DOC) [file pone.0094886.s002.doc]

**Table S2.** STr: specimen treatment; CFU: colony forming units assessed after 24 h of culture of fresh suspensions; HRM: High resolution melting real-time PCR; PK: proteinase K at 56° for 10 min, cooling at -18C for 5 min and extraction with magnetic beads; Ly: Lyticase at 56° for 10 min, cooling at -18C for 5 min and extraction with magnetic beads; IC: internal control; pIC: IC tested with Taqman real-time PCR; HRM: High resolution melting real-time PCR; FilamUn: forward primer for filamentous *Fungi*; CandUn: forward primer for yeasts; FungUn: Universal reverse primer for all *Fungi*; Anig: *Aspergillus niger*; Calb: *Candida albicans*, Fus: *Fusarium solani*; NC: negative control; -: Negative, Ct > 42; SpAmp: amplification cycles leading to a plateau (amplification of 100%); Id: Fungi identification according to family after melting curve analysis; Y: Yes, the shape of the melting curve corresponds to searched species according to strain profiles; N: melting curve analysis did not allow fungal identification; NA: not analyzed; MP: MagNA Pure compact (Roche) without pretreatment; QIA (QIAamp DNA Minikit QIAgen) without pretreatment.

| Agent | [CFU/ml] | STr | pIC * | HRM | | | |
| --- | --- | --- | --- | --- | --- | --- | --- |
|  |  |  |  | Set of primers | | | |
|  |  |  |  | CandUn+ FungUn | | FilamUn+FungUn | |
|  |  |  |  | SpAmp | Id | SpAmp | Id |
| Anig | 10^4^ | PK | 31.7 | Y | N | Y | N |
|  |  | LY | 30.1 | Y | N | Y | Y |
|  |  | PK+Ly | 29.9 | Y | N | Y | Y |
|  | 10^3^ | PK | 30.5 | Y | N | Y | N |
|  |  | LY | 29.7 | Y | N | Y | Y |
|  |  | PK+Ly | 30.1 | Y | N | Y | Y |
|  | 10^2^ | PK | 29.8 | N | NA | Y | N |
|  |  | LY | 29.2 | Y | N | Y | Y |
|  |  | PK+Ly | 29.1 | Y | N | Y | Y |
|  |  | MP | 30.0 | N | N | N | N |
|  |  | QIA | 32.2 | N | N | N | N |
| C.alb | 10^4^ | PK | 30.1 | Y | Y | Y | N |
|  |  | LY | 28.9 | Y | Y | Y | N |
|  |  | PK+Ly | 29.4 | Y | Y | Y | N |
|  | 10^3^ | PK | 28.7 | Y | Y | Y | N |
|  |  | LY | 29.2 | Y | Y | Y | N |
|  |  | PK+Ly | 29.8 | Y | Y | Y | N |
|  | 10^2^ | PK | 29.1 | Y | N | N | NA |
|  |  | LY | 29.0 | Y | Y | Y | N |
|  |  | PK+Ly | 29.6 | Y | Y | Y | N |
|  |  | MP | 32.0 | Y | N | N | N |
|  |  | QIA | 30.8 | Y | N | N | N |
| Fus | 10^4^ | PK | 30.0 | Y | N | Y | N |
|  |  | LY | 29.9 | Y | N | Y | Y |
|  |  | PK+Ly | 30.2 | Y | N | Y | Y |
|  | 10^3^ | PK | 31.1 | Y | N | Y | N |
|  |  | LY | 29.7 | Y | N | Y | Y |
|  |  | PK+Ly | 30.3 | Y | N | Y | Y |
|  | 10^2^ | PK | 30.0 | N | NA | Y | N |
|  |  | LY | 29.9 | Y | N | Y | Y |
|  |  | PK+Ly | 30.7 | Y | N | Y | Y |
|  |  | MP | 28.9 | N | N | Y | N |
|  |  | QIA | 32.1 | N | N | N | N |
